# Description and evaluation of an ultra-processed food consumption score for children

**DOI:** 10.11606/s1518-8787.2025059005816

**Published:** 2025-03-13

**Authors:** Anna Müller, Caroline dos Santos Costa, Romina Buffarini, Juliana dos Santos Vaz, Marlos Rodrigues Domingues, Pedro Curi Hallal, Janaina Vieira dos Santos Motta

**Affiliations:** I Universidade Federal de Pelotas Faculdade de Medicina Programa de Pós-Graduação em Epidemiologia Pelotas RS Brasil Universidade Federal de Pelotas. Faculdade de Medicina. Programa de Pós-Graduação em Epidemiologia. Pelotas, RS, Brasil; II Universidade Federal do Rio Grande Faculdade de Medicina Pós-Graduação em Ciências da Saúde Rio Grande RS Brasil Universidade Federal do Rio Grande. Faculdade de Medicina. Pós-Graduação em Ciências da Saúde. Rio Grande, RS, Brasil; III Universidade Federal de Pelotas Faculdade de Nutrição Programa de Pós-Graduação em Nutrição e Alimentos Pelotas RS Brasil Universidade Federal de Pelotas. Faculdade de Nutrição. Programa de Pós-Graduação em Nutrição e Alimentos. Pelotas, RS, Brasil; IV Universidade Federal de Pelotas Escola Superior de Educação Física Programa de Pós-Graduação em Educação Física Pelotas RS Brasil Universidade Federal de Pelotas. Escola Superior de Educação Física. Programa de Pós-Graduação em Educação Física. Pelotas, RS, Brasil

**Keywords:** Food intake, Processed Food, Child Nutrition, Surveys and Questionnaires, Validation Study

## Abstract

**OBJECTIVE:**

To present a screener for consumption of ultra-processed foods for children in early childhood, evaluating the ability of the score generated by this screener to reflect the participation of ultra-processed foods in children’s diets.

**METHODS:**

This study was conducted with a convenience subsample of the 2015 Pelotas Birth Cohort (n = 365). The mothers of the participating children answered a food consumption questionnaire the day before the interview (screener) containing 16 subgroups of ultra-processed foods, followed by a traditional 24-hour food recall (24hR). Each participant’s ultra-processed food consumption score corresponded to the number of food subgroups consumed and the percentage of energy derived from the participation of ultra-processed foods in the diet on the same day was calculated from the answers in the 24hR. The association between the score and the percentage of energy from ultra-processed foods was tested using linear regression models. The degree of agreement between the classification of participants according to approximate fifths of the percentage of calories from ultra-processed foods and according to the score intervals was assessed using the Pabak index.

**RESULTS:**

The average percentage share of ultra-processed foods in the total caloric value of the diet, calculated using the 24hR, was directly and significantly associated with an increase in the ultra-processed food consumption score. There was substantial agreement between the ranges of the ultra-processed food consumption score obtained by the screener and the approximate fifths of the share of ultra-processed foods in the diet calculated by the 24hR (Pabak index = 0.65).

**CONCLUSIONS:**

The ultra-processed food consumption score, obtained from an ultra-processed food consumption screener, a practical and agile instrument, is capable of reflecting the participation of ultra-processed foods in children’s diets, with regard to the 2015 Pelotas Birth Cohort.

## INTRODUCTION

According to the Nova classification^1^, ultra-processed foods are defined as industrial formulations resulting from a sequence of processes, including fractionation of whole foods into substances, modification and/or recombination of these substances, as well as the use of cosmetic additives and attractive packaging. These products are highly durable, cost-effective because they use low-cost ingredients, ready-to-eat, hyper-palatable, and have the potential to replace all other food groups.^1^

Excessive consumption of ultra-processed foods has been associated with a general deterioration in the nutritional quality of diets, since it is directly linked to excess energy consumption and increased consumption of free sugars, total fats, and saturated fats and decreased consumption of fiber, proteins, and vitamins^2,3^. Due to the formulation and characteristics of these foods, their consumption is related to adverse effects on the intestinal microbiota and body composition^4^, as well as a series of metabolic alterations, including increased blood pressure and levels of cholesterol, triglycerides, and serum lipids.^5^

A growing number of epidemiological studies are using Nova as a basis for classifying foods with food consumption data collected from food frequency questionnaires, food records, and 24-hour recall, which allow the calculation of the calorie percentage of the diet from ultra-processed foods. However, although traditional instruments for collecting food consumption data provide a greater amount of data for analyzing food consumption, they have important disadvantages, such as the need for greater financial and human resources for their application^6^, which can make it difficult to assess and monitor the consumption of ultra-processed foods in many contexts or populations.

Considering this scenario, new short, quick, and easy-to-apply tools have been developed and tested, making it possible to obtain an indirect measure of the share of ultra-processed foods in the diet, based on the Nova classification. One of these tools is the Nova ultra-processed food consumption screener (NovaScreener)^6,7^, which consists of obtaining information on food consumption the previous day (yes or no) from a list of ultra-processed food subgroups, which allows a score to be calculated, called the Nova ultra-processed food consumption score, where each positive response corresponds to one point on the score. This tool and others like it have been applied in population surveys of adults, such as the Surveillance of Risk and Protective Factors for Chronic Diseases by Telephone Survey (Vigitel)^8^and NutriNet Brasil^11^ and used to reflect the participation of ultra-processed foods in the diet. However, similar tools specific to the child population have not yet been proposed.

In view of this, the aim of this study was to present a screener for consumption of ultra-processed foods for children in early childhood, evaluating the ability of the score generated by this screener, compared to a 24-hour recall, to reflect the participation of ultra-processed foods in children’s diets, in the context of the 2015 Pelotas Birth Cohort.

## METHODS

### Pelotas Birth Cohort - 2015

The 2015 Birth Cohort is a longitudinal study that recruited all live births in that year in the city of Pelotas, RS, a medium-sized city in southern Brazil, and is the most recent of the four cohorts carried out in the city (1982, 1993, 2004, and 2015). The 2015 cohort consisted of 4,333 children born in the city’s hospitals to mothers living in urban areas. Discounting a loss and refusal rate of 1.3% and 54 stillbirths, the final sample of the 2015 Birth Cohort corresponded to 4,275 children. For perinatal follow-up, mothers were interviewed between 24 and 48 hours after giving birth. Subsequently, mothers and children were interviewed at various times (three and 12 months; two, four, and 6-7 years of the child’s age) to assess various socioeconomic, health, and nutritional characteristics of the participants. The follow-up rate for the 2015 Birth Cohort at 6-7 years of age was 90.5% (n = 3,867). More information is available in the 2015 Cohort profile.^12^

### Data collection

This study was carried out with a convenience sub-sample of mothers and children participating in the 6-7 year follow-up of the 2015 Pelotas Birth Cohort. To be eligible for the study, mothers had to respond positively to a filter question: “Can you describe <CHILD>’s diet yesterday?”. In this sub-sample, information was collected on the consumption of ultra-processed foods the day before the interview. The data was collected between November 2021 and November 2022, at the Dr. Amilcar Gigante Health Research Center, in a clinic specially set up to attend to the children and mothers of the Pelotas birth cohorts, by a team of trained interviewers, using the REDCap software*.* ^13^

The first instrument to be applied was the tool under evaluation - the ultra-processed food consumption screener for children. Immediately after the end of the interview, a traditional 24-hour recall (24hR) of the child’s diet was administered by the same interviewer.

### Ultra-processed Food Consumption Screener

The tracer for assessing consumption of ultra-processed foods the day before was developed for use in the 6-7 year-old follow-up of the 2015 Birth Cohort. This tool was based on the one proposed by the Center for Epidemiological Research in Nutrition and Health (Nupens)^7^ and the new module of questions on food consumption from the Vigitel System (2018-2019)^10^, both aimed at adults and built on consumption data from the Household Budget Survey (POF 2008-2009)^14^. When constructing the instrument for the 2015 Birth Cohort, some adaptations were made, based on empirical knowledge about the consumption habits of children in Pelotas, mainly in relation to the examples of the items.

The ultra-processed food consumption screener comprises 16 items or subgroups of these: packaged snacks (chips); biscuits/sweet cookies, stuffed cookies, or packaged muffins; instant noodles (like cup noodles) and packaged soup; chocolate, ice cream/popsicles, or industrialized desserts; candies, lollipops, chewing gum or jelly; margarine; buns, hot dogs buns, or hamburger buns (packaged bread); nuggets, industrialized breaded chicken, hamburgers, and sausages; frozen fries or fries from fast-food chains; ham, mortadella, or salami; ready-made or frozen dishes such as pizzas, lasagna, *escondidinho*; boxed or powdered juice, boxed coconut water or guarana/gooseberry syrups; chocolate drink or chocolate powder added to milk; flavored yogurt or milk drink; soft drinks; breakfast cereals. This instrument assesses food consumption on the day before the interview (no/yes) and served as the basis for constructing the ultra-processed food consumption score, built for each participant from the sum of the ultra-processed food subgroups consumed, among the 16 listed, thus ranging from 0 to 16.

### 24-Hour Recall

The 24hR was administered following three steps. Firstly, the mother was informed about the purpose of the report and the recall period (the day before the interview). Next, the mother was asked to give a detailed account of all the foods and liquids consumed by the child, including the portion size, homemade measure, preparation (homemade, bought frozen, etc.) and repetitions. Finally, the interview ended with the interviewer reading out all the food reported by the mother, in order to review and encourage the reporting of forgotten and/or omitted foods. To help with the reporting of quantities and home measures, the table of home measures from the National Survey of Child Nutrition (ENANI)^15^ was used. The 24hR data was collected between Tuesdays and Saturdays, in order to include estimates of the child’s diet only on weekdays, when consumption tends to be habitual.

Each consumption item reported in household measures in the 24hR was transformed into grams and converted into calories using the Brazilian Table of Food Composition (TACO)^16^. Subsequently, the foods were classified into four groups, according to the Nova classification^1^. Finally, the total number of calories consumed by the participants was calculated, and 10 participants with a daily calorie intake greater than +3 standard deviations from the mean (8,031 kcal) were excluded^17^. Calories from ultra-processed foods and the percentage energy contribution of ultra-processed foods in relation to the total were also calculated.

### Sociodemographic variables

The sociodemographic variables assessed were: child’s sex, mother’s self-reported skin color (white, black, brown), collected at perinatal follow-up; mother’s age in years (categorized into 20-34, 35-39, and 40 years or more), mother’s schooling in complete years of study (categorized into 0-4, 5-8, 9-11, and 12 years or more) and family income in Brazilian currency (collected as the sum of the individual incomes of all the residents of the house and then categorized into quintiles), collected at the 6-7 year follow-up.

### Data Analysis

Initially, the sample was described according to sociodemographic characteristics, with the crude (n) and relative (%) frequencies of each variable presented. The proportion (%) of positive responses in the sample for each item or subgroup of ultra-processed foods was also presented, as well as the distribution of the sample according to each score.

To assess the score’s ability to reflect the share of ultra-processed foods in children’s diets, linear regression was first used to assess the average number of calories coming from ultra-processed foods according to the variation in the score, expressed in its original form, but with the highest score being 10 or more due to the small sample size in scores from 11 upwards, as well as intervals corresponding to (approximate) fifths of the score distribution (intervals 0-3, 4, 5-6, 7, and ≥ 8).

The degree of agreement between the classification of participants according to approximate fifths of the percentage of calories from ultra-processed foods and according to the score intervals was assessed using the Pabak (prevalence-adjusted and bias-adjusted kappa) index. The index indicates almost perfect agreement when it is greater than 0.80, substantial when it is between 0.61 and 0.80, moderate between 0.41 and 0.60, reasonable between 0.21 and 0.40 and weak when it is equal to or less than 0.20^18^. To do this, approximate fifths of the percentage of energy coming from ultra-processed foods were created, based on the proportions of the approximate fifths of the ultra-processed food consumption score.

All the analyses were carried out in the Stata 17.0 software and the Pabak index was calculated in the RStudio software using the ˜irrCAC˜ statistical package, applying quadratic weights using the “quadratic.weights” function. The 95% confidence intervals were also calculated in RStudio. The weighting method used was chosen with the aim of giving more weight to the closest categories.

### Ethical aspects

The 2015 Birth Cohort study protocol was reviewed and approved by the research ethics committee of the School of Physical Education of the Federal University of Pelotas (0-4 year follow-ups: 26746414.5.0000.5313; 6-year follow-ups: 51789921.1.0000.5317) and the signing of the Informed Consent Form (ICF) was obtained before each interview.

## RESULTS

The final study sample consisted of 365 children, the majority of whom were female (51.8%), white (66.1%), the daughters of mothers aged between 20 and 34 (54.3%) and with 12 years or more of schooling (34.5%). Table 1 describes the sociodemographic characteristics of the sample.

**Table 1:** Distribution of children from the 2015 Pelotas Birth Cohort according to sociodemographic variables. Brazil. (n = 365a).

Features	n	%
Sex		
Male	176	48.2
Female	189	51.8
Skin color		
White	238	66.1
Black	63	17.5
Brown	59	16.4
Maternal age (years)		
20-34	197	54.3
35-39	89	24.5
≥ 40	77	21.2
Maternal schooling (complete years of study)		
0-4	13	4.3
5-8	94	30.6
9-11	94	30.6
≥ 12	106	34.5
Family income in reais^b^		
Q1 (poorest)	83	23.1
Q2	90	25.0
Q3	60	16.7
Q4	57	15.8
Q5 (richest)	70	19.4

Q: quintile; m: median

^a^ Ignored observations: 5 (1.4%) for skin color, 2 (0.5%) for maternal age, 58 (15.9%) for maternal schooling, and 5 (1.4%) for family income.

^b^ Family income in reais: Q1 (m = 900); Q2 (m = 2,000); Q3 (m = 2,950); Q4 (m = 4,500); Q5 (10,250).

The figure shows the frequency of consumption on the day before the interview of each of the 16 subgroups of ultra-processed foods included in the screener. Two out of three children consumed biscuits/sweet cookies, stuffed cookies or packaged muffins (66%) and half of the sample consumed chocolate drinks or chocolate powder (added to milk) (51.8%), as well as buns, hot dog buns or hamburger buns (packaged bread) (51.5%) and margarine (49.5%). Around a third of children consumed sweets, lollipops, chewing gum, or jelly (39.5%), ham, mortadella, or salami (37.5%), boxed or powdered juice, boxed coconut water, or syrups (guarana/gooseberry) (37,5%), salty snacks (chips) (34%), soft drinks (any type) (34%), flavored yogurt or dairy drinks (32.3%), and chocolate, ice cream/popsicles, or industrialized desserts (31.2%). The other food subgroups were consumed by less than 15% of the children in the sample. The average percentage of ultra-processed foods consumed was 49.4%.

**Figure:**
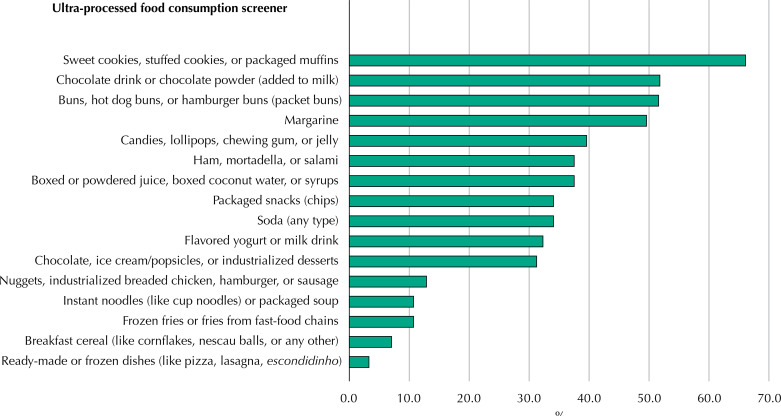
Frequency (%) of consumption of foods included in the ultra-processed food consumption screener on the day prior to the interview,. 2015 Pelotas Birth Cohort, Brazil. (n = 365).

The distribution of the ultra-processed food consumption score (number of subgroups consumed on the day before the interview) is shown in Table 2. The score ranged from 0 to 10 or more foods consumed, with scores 4, 5, and 6 being the most frequent (19.7%, 16.4%, and 15.9%, respectively). The average percentage share of ultra-processed foods in the total caloric value of the diet, calculated using the 24hR, was directly and significantly associated with an increase in the ultra-processed food consumption score (Table 2).

**Table 2:** Share of ultra-processed foods according to the score in a 1-day food recall at 6 years of age. 2015 Pelotas Birth Cohort, Brazil. (n = 365).

Ultra-processed food consumption score	Sample	Total energy intake	Share of ultra-processed foods in the diet (% of total energy)
	n (%)	Average in Kcal	Average (95% CI)
1	11 (3.0)	1,994.3	29.9 (16.3-43.6)
2	33 (9.1)	1,967.1	41.0 (33.1-48.8)
3	31 (8.5)	2,444.6	41.5 (39.4 - 55.6)
4	72 (19.7)	2,330.4	44.5 (39.2-49.8)
5	60 (16.4)	2,338.2	49.6 (43.8-55.5)
6	58 (15.9)	2,416.9	54.9 (48.9-60.8)
7	39 (10.7)	2,443.6	50.8 (43.5-58.0)
8	29 (7.9)	2,685.6	60.4 (52.0-68.8)
9	19 (5.2)	2,941.9	57.2 (46. 9-67.6)
≥ 10	13 (3.6)	2,436.7	53.9 (41.3-66.4)^a^
1-3	75 (20.5)	2,168.5	41.1 (38.8-47.3)
4	72 (19.7)	2,330.5	44.5 (39.1-49.8)
5-6	118 (32.3)	2,376.9	52.2 (48.0-56.4)
7	39 (10.7)	2,443.6	50.8 (43.5-58.0)
≥ 8	61 (16.7)	2,712.4	58.0 (52.2-63.8)^a^

95% CI: 95% confidence interval;

^a^ Convenience sub-sample.

^b^ P-value for linear trend < 0.001.

R2 of the model: 0.0745

Table 3 shows the simultaneous distribution according to the approximate fifths of the score and the share of ultra-processed foods in the diet. From this distribution of the sample, substantial agreement was observed between the ranges of the ultra-processed food consumption score obtained by the screener and the approximate fifths of the share of ultra-processed foods in the diet calculated by the 24hR (Pabak index of 0.65, 95%CI: 0.48-0.83).

**Table 3:** Agreement between the distribution (%) of ultra-processed foods in a 1-day 24-hour recall and quintiles of the NOVA score at 6 years of age. 2015 Pelotas Birth Cohort, Brazil. (n = 365).

Fifths of the share of ultra-processed foods in the diet (% of total calories)	Fifths of the Nova ultra-processed food consumption score						
	1-3	4	5-6	7	≥ 8	Total	PABAK Index^a^ (95%CI)
Q1 (< 26.6)	6	4.9	6	1.1	2.5	20.5	0.65 (0.48-0.83)
Q2 (26.7-44.1)	6	3.3	6	1.9	2.5	19.7	
Q3 (44.2-64.5)	5.8	5.8	11.8	3	6	32.4	
Q4 (64.6-74.8)	1.4	2.5	3.8	1.4	1.6	10.7	
Q5 (≥ 74.9)	1.4	3.3	4.6	3.3	4.1	16.7	
Total	20.6	19.8	32.2	10.7	16.7	100	

^a^ Pabax index (kappa adjusted for prevalence and bias)

## DISCUSSION

This study presented a tracer for consumption of ultra-processed foods proposed for children in early childhood and evaluated the ability of the score generated from this tracer to reflect the participation of ultra-processed foods in children’s diets, in the context of the 2015 Birth Cohort in the city of Pelotas, RS. The results showed that the ultra-processed food consumption score, obtained from the food consumption screener, showed a direct and linear association with the percentage of energy derived from ultra-processed food intake, generated from the 24-hour recall. The results also showed substantial agreement between the classification of participants according to the score distribution intervals and the approximate fifths of the distribution of the percentage of ultra-processed foods in the diet.

The results obtained in this study are in line with the findings of a similar study on a version of the score used for adults^19^, indicating that it is possible to reflect the participation of ultra-processed foods in the diet from the data obtained by the ultra-processed food consumption screener. However, there are still few studies evaluating the consumption of ultra-processed foods by children, especially in early childhood, the period from zero to six years of age, probably due to the difficulty in measuring the consumption of these foods, without the need for greater financial and human resources for data collection.

A study carried out in the 2015 Birth Cohort^20^, at the age of two, showed that around 2% of the followed-up children (n = 4,275) did not consume any subgroup of ultra-processed foods habitually. In the present study, carried out with a sub-sample of the same cohort, all the children monitored consumed at least one subgroup of ultra-processed foods. Another study carried out in the same cohort assessed the consumption of ultra-processed foods at two and four years of age and showed that the proportions of consumption of eight of the nine food subgroups assessed increased with age^21^. This scenario of high consumption is worrying because, according to the recommendations of the food guides for children under two^22^ and for the Brazilian population^23^, ultra-processed foods should not be consumed before the first two years and should be avoided in the other stages of life. In this sense, we highlight the importance of new quick, short and easy-to-use instruments that facilitate the collection of dietary data and allow more studies to be carried out on the consumption of ultra-processed foods.

The average percentage of ultra-processed foods consumed in this study was 49.4%. Possibly, the high consumption of these foods by the sample studied did not allow the intervals of the ultra-processed food consumption score to be well determined, showing better agreement between the methods in the third quintile. However, although the percentages of agreement were not as high, there was also agreement in the adjacent fifths, thus corroborating the substantial agreement found between the methods.

This study has a limitation regarding the ultra-processed food consumption screener. The instrument was built based on the instruments used in Vigitel^10^ and Nutrinet Brasil^11^, both proposed based on data from the POF^13^ for adults. Although it was based on the Nova ultra-processed food consumption screener (NovaScreener)^7^, our questionnaire didn’t use the same methodology, since we don’t have data from specific national surveys for children in the age group evaluated. Another limitation is that, despite the sufficient sample size to test agreement between two instruments^24^ (n = 365), the sub-sample of a birth cohort did not allow us to assess the instrument’s capacity for subgroups of the population, based on variables such as family income and maternal schooling, for example.

## CONCLUSIONS

The ultra-processed food consumption score, obtained from an ultra-processed food consumption screener, a practical and agile tool, is capable of reflecting the participation of ultra-processed foods in children’s diets, as far as the 2015 Pelotas Birth Cohort is concerned. Further studies evaluating this or other ultra-processed food consumption screeners in nationally representative samples of children will be important to strengthen the use of these tools in the child population.
