# Erratum

**DOI:** 10.11606/s1518-8787.2025059005816err

**Published:** 2025-06-11

In article Description and evaluation of an ultra-processed food consumption score for children, with DOI number https://doi.org/10.11606/s1518-8787.2025059005816, published in the journal Revista de Saúde Pública, v. 59, e5 on page 9,

**Where it reads:**

11. NUTRINET Brasil. Relatório do estudo Nutrinet Brasil: avaliaçâo do impacto da alimentaçâo na saúde dos brasileiros. Sâo Paulo: Universidade de Sâo Paulo; 2021 [cited 2022 Oct 1]. Available from: http://www.nutrinetsaude.com.br/relatorio2021.

**It should read:**

11. Nutrinet Brasil. Nutrinet Brasil [Internet]. Sâo Paulo: Faculdade de Saúde Publica da USP; 2025 [cited 2022 Oct 1]. Available from: https://nutrinetbrasil.fsp.usp.br/

