# Supplementary material for: Description and evaluation of an ultra-processed food consumption score for children
Source: Rev Saude Publica. 2025 Mar 13;59:e5. doi: 10.11606/s1518-8787.2025059005816 (PMC11967337; doi:10.11606/s1518-8787.2025059005816)
Supplement: Translation

# Translation: Descrição e avaliação de um escore de consumo de alimentos ultraprocessados para crianças

**Authors:** Anna Müller, Caroline dos Santos Costa, Romina Buffarini, Juliana dos Santos Vaz, Marlos Rodrigues Domingues, Pedro Curi Hallal, Janaina Vieira dos Santos Motta

**Affiliations:** I Brasil Universidade Federal de Pelotas. Faculdade de Medicina. Programa de Pós-Graduação em Epidemiologia. Pelotas, RS, Brasil; II Brasil Universidade Federal do Rio Grande. Faculdade de Medicina. Pós-Graduação em Ciências da Saúde. Rio Grande, RS, Brasil; III Brasil Universidade Federal de Pelotas. Faculdade de Nutrição. Programa de Pós-Graduação em Nutrição e Alimentos. Pelotas, RS, Brasil; IV Brasil Universidade Federal de Pelotas. Escola Superior de Educação Física. Programa de Pós-Graduação em Educação Física. Pelotas, RS, Brasil

**Keywords:** Ingestão de Alimentos, Alimento Processado, Nutrição da Criança, Inquéritos e Questionários, Estudo de Validação

## Abstract

**OBJETIVO:**

Apresentar um rastreador para consumo de alimentos ultraprocessados para crianças na primeira infância, avaliando a capacidade do escore gerado por esse rastreador de refletir a participação de alimentos ultraprocessados na alimentação das crianças.

**MÉTODOS:**

Estudo realizado com uma subamostra de conveniência da Coorte de Nascimentos de Pelotas de 2015 (n = 365). As mães das crianças participantes responderam a um questionário de consumo alimentar no dia anterior à entrevista (rastreador) contendo 16 subgrupos de alimentos ultraprocessados, seguido por um recordatório alimentar tradicional de 24 horas (R24h). O escore de consumo de alimentos ultraprocessados de cada participante correspondeu ao número de subgrupos de alimentos consumidos e o percentual de energia proveniente da participação dos alimentos ultraprocessados na alimentação do mesmo dia foi calculado a partir das respostas do R24h. A associação entre o escore e o percentual de energia proveniente de alimentos ultraprocessados foi testada por modelos de regressão linear. A avaliação do grau de concordância entre a classificação dos participantes segundo quintos aproximados do percentual de calorias de alimentos ultraprocessados e segundo os intervalos do escore foi feita a partir do índice Pabak.

**RESULTADOS:**

O percentual médio de participação de alimentos ultraprocessados no valor calórico total da dieta, calculado com base no R24h, foi direta e significativamente associado com o aumento do escore de consumo de alimentos ultraprocessados. Foi observada concordância substancial entre os intervalos do escore de consumo de alimentos ultraprocessados, obtido pelo rastreador, e os quintos aproximados da participação de alimentos ultraprocessados na dieta, calculada pelo R24h (índice Pabak = 0,65).

**CONCLUSÕES:**

O escore de consumo de alimentos ultraprocessados, obtido a partir de um rastreador de consumo de alimentos ultraprocessados, um instrumento prático e ágil, é capaz de refletir a participação de alimentos ultraprocessados na alimentação das crianças, no que diz respeito à Coorte de Nascimentos de Pelotas de 2015.

## INTRODUÇÃO

De acordo com a classificação Nova^1^, alimentos ultraprocessados são definidos como formulações industriais resultantes de uma sequência de processos, incluindo fracionamento de alimentos inteiros em substâncias, modificação e/ou recombinação dessas substâncias, bem como uso de aditivos cosméticos e embalagens atrativas. Esses produtos são altamente duráveis, rentáveis por utilizarem ingredientes de baixo custo, prontos para o consumo, hiperpalatáveis e com potencial para substituir todos os outros grupos de alimentos^1^.

O consumo excessivo de alimentos ultraprocessados tem sido associado a uma deterioração geral da qualidade nutricional das dietas, uma vez que está diretamente ligado ao consumo em excesso de energia e ao aumento do consumo de açúcares livres, gorduras totais e gorduras saturadas e diminuição do consumo de fibras, proteínas e vitaminas^2,3^. Devido à formulação e às características desses alimentos, seu consumo está relacionado a efeitos adversos na microbiota intestinal e composição corporal^4^, bem como a uma série de alterações metabólicas, incluindo o aumento da pressão arterial e dos níveis de colesterol, triglicerídeos e lipídio sérico^5^.

É crescente o número de estudos epidemiológicos que utilizam a Nova como base para a classificação dos alimentos com dados de consumo alimentar coletados a partir de questionários de frequência alimentar, registros alimentares e recordatórios de 24 horas, os quais permitem o cálculo do percentual calórico da dieta proveniente de alimentos ultraprocessados. Entretanto, apesar de instrumentos tradicionais para coleta de dados do consumo alimentar fornecerem maior quantidade de dados para analisar o consumo alimentar, eles possuem desvantagens importantes, como a necessidade de maiores recursos financeiros e humanos para sua aplicação^6^, o que pode dificultar a avaliação e o monitoramento do consumo de alimentos ultraprocessados em muitos contextos ou populações.

Considerando esse cenário, novos instrumentos curtos, rápidos e fáceis de serem aplicados têm sido desenvolvidos e testados, permitindo obter uma medida indireta da participação dos alimentos ultraprocessados na alimentação, com base na classificação Nova. Uma dessas ferramentas é o rastreador Nova de consumo de alimentos ultraprocessados (NovaScreener)^6,7^, o qual consiste na obtenção de informações sobre o consumo alimentar no dia anterior (consumiu sim ou não) de uma lista de subgrupos de alimentos ultraprocessados, que permitem o cálculo de um escore, chamado escore Nova de consumo de alimentos ultraprocessados, onde cada resposta positiva corresponde a um ponto no escore. Esse instrumento e outros similares têm sido aplicados em inquéritos populacionais realizados com indivíduos adultos, como a Vigilância de Fatores de Risco e Proteção para Doenças Crônicas por Inquérito Telefônico (Vigitel)^8^e o NutriNet Brasil^11^, e utilizados para refletir a participação de alimentos ultraprocessados na dieta. Entretanto, ferramentas similares específicas para a população infantil ainda não foram propostas.

Diante disso, o objetivo deste estudo foi apresentar um rastreador para consumo de alimentos ultraprocessados para crianças na primeira infância, avaliando a capacidade do escore gerado por este rastreador, em comparação com um recordatório de 24h, de refletir a participação de alimentos ultraprocessados na alimentação das crianças, no contexto da Coorte de Nascimentos de Pelotas de 2015.

## MÉTODOS

### Coorte de Nascimentos de Pelotas - 2015

A Coorte de Nascimentos de 2015 é um estudo longitudinal que recrutou todos os nascidos vivos no referido ano na cidade de Pelotas, RS, cidade de médio porte do sul do Brasil, sendo a mais recente das quatro coortes realizadas na cidade (1982, 1993, 2004 e 2015). A Coorte de 2015 foi formada por 4.333 crianças nascidas nos hospitais da cidade e filhas de mães residentes na área urbana. Descontando uma taxa de perdas e recusas de 1,3% e 54 natimortos, a amostra final da Coorte de Nascimentos de 2015 correspondeu a 4.275 crianças. Para o acompanhamento perinatal, as mães foram entrevistadas entre 24 e 48 horas após o parto. Posteriormente, as mães e crianças foram entrevistadas em diversas ocasiões (três e 12 meses; dois, quatro e 6–7 anos de idade da criança), para avaliar diversas características socioeconômicas, de saúde e nutricionais dos participantes. A taxa de acompanhamento da Coorte de Nascimentos de 2015 aos 6–7 anos de idade foi de 90,5% (n = 3.867). Mais informações encontram-se disponíveis no perfil da Coorte 2015^12^.

### Coleta de Dados

Este estudo foi realizado com uma subamostra de conveniência de mães e crianças participantes do acompanhamento dos 6–7 anos de idade da Coorte de Nascimentos de Pelotas de 2015. Para ser elegível ao estudo, as mães deveriam responder positivamente a uma pergunta filtro: “A senhora sabe descrever a alimentação da <CRIANÇA> no dia de ontem?”. Nesta subamostra, foram coletadas informações sobre o consumo de alimentos ultraprocessados no dia anterior à entrevista. Os dados foram coletados entre novembro de 2021 e novembro de 2022, no Centro de Pesquisa em Saúde Dr. Amilcar Gigante, em uma clínica especialmente montada para atender as crianças e mães integrantes das coortes de nascimento de Pelotas, por uma equipe de entrevistadoras treinadas, através do *software* REDCap^13^.

O primeiro instrumento a ser aplicado foi a ferramenta em avaliação – rastreador do consumo de alimentos ultraprocessados para crianças. Imediatamente após o final da entrevista, um recordatório tradicional de 24 horas (R24h) sobre a alimentação da criança foi aplicado, pela mesma entrevistadora.

### Rastreador de Consumo de Alimentos Ultraprocessados

O rastreador para avaliação do consumo de alimentos ultraprocessados no dia anterior foi desenvolvido para ser utilizado no acompanhamento dos 6–7 anos de idade da Coorte de Nascimentos de 2015. Esse instrumento foi baseado no proposto pelo Núcleo de Pesquisas Epidemiológicas em Nutrição e Saúde (Nupens)^7^ e no novo módulo de questões sobre o consumo alimentar do Sistema Vigitel (2018–2019)^10^, ambos voltados para indivíduos adultos e construídos com base nos dados de consumo da Pesquisa de Orçamentos Familiares (POF 2008–2009)^14^. Na construção do instrumento para a Coorte de Nascimentos de 2015, algumas adaptações foram feitas, a partir do conhecimento empírico sobre os hábitos de consumo das crianças pelotenses, principalmente em relação aos exemplos dos itens.

O rastreador de consumo de alimentos ultraprocessados compreende 16 itens ou subgrupos desses: salgadinhos de pacote (tipo *chips*); biscoito/bolacha doce, bolacha recheada ou bolinho de pacote; macarrão instantâneo (do tipo miojo) e sopa de pacote; chocolate, sorvete/picolé ou sobremesa industrializada; balas, pirulitos, chicletes ou gelatina; margarina; pão de forma, de cachorro-quente ou de hambúrguer (pão de pacote); *nuggets*, frango empanado industrializado, hambúrguer e salsicha; batata frita tipo congelada ou de redes de *fast-food*; presunto, mortadela ou salame; pratos prontos ou congelados, como pizzas, lasanha, escondidinho; suco de caixinha ou em pó, água de coco em caixinha ou xaropes de guaraná/groselha; bebida achocolatada ou achocolatado em pó adicionado ao leite; iogurte com sabor ou bebida láctea; refrigerante; cereal matinal. Esse instrumento avalia o consumo alimentar no dia anterior à entrevista (não/sim) e serviu de base para a construção do escore de consumo de alimentos ultraprocessados, construído para cada participante a partir da somatória de subgrupos de alimentos ultraprocessados consumidos, dentre os 16 listados, variando, dessa forma, de 0 a 16.

### Recordatório de 24 Horas

O R24h foi aplicado seguindo três passos. Primeiramente, a mãe foi orientada quanto ao objetivo do relato e o período recordatório (dia anterior à entrevista). Em seguida, foi solicitado o relato detalhado de todos os alimentos e líquidos consumidos pela criança, incluindo a indicação do tamanho da porção, medida caseira, preparo (feito em casa, comprado pronto congelado etc.) e repetições. Por fim, a entrevista foi finalizada com a leitura da entrevistadora de todos os alimentos relatados pela mãe, com o intuito de revisar e estimular o relato de alimentos esquecidos e/ou omitidos. Para auxiliar no relato das quantidades e medidas caseiras, foi utilizada a tabela de medidas caseiras do Estudo Nacional de Alimentação e Nutrição Infantil (Enani)^15^. Os dados do R24h foram coletados entre terças-feiras e sábados, a fim de incluir estimativas da alimentação da criança apenas em dias de semana, quando o consumo tende a ser habitual.

Cada item de consumo informado em medidas caseiras no R24h foi transformado em gramas e convertido em calorias, utilizando-se a Tabela de Composição de Alimentos Consumidos no Brasil (TACO)^16^. Posteriormente, os alimentos foram classificados em quatro grupos, de acordo com a classificação Nova^1^. Por fim, o total de calorias consumidas pelos participantes foi calculado, sendo excluídos 10 participantes com consumo calórico diário superior a +3 desvios-padrão da média (8.031 kcal)^17^. Foram também calculadas as calorias provenientes de alimentos ultraprocessados e o percentual de contribuição energética de alimentos ultraprocessados em relação ao total.

### Variáveis Sociodemográficas

As variáveis sociodemográficas avaliadas foram: sexo da criança, cor da pele da mãe autorreferida (branca, preta, parda), coletadas no acompanhamento perinatal; idade da mãe em anos (categorizada em 20–34, 35–39 e 40 anos ou mais), escolaridade da mãe em anos completos de estudo (categorizada em 0–4, 5–8, 9–11, 12 anos ou mais) e renda familiar em moeda brasileira (coletada como somatória das rendas individuais de todos os moradores da casa e logo categorizada em quintis), coletadas no acompanhamento dos 6–7 anos de idade.

### Análise de Dados

Inicialmente, a amostra foi descrita segundo características sociodemográficas, com apresentação das frequências bruta (n) e relativa (%) de cada variável. A proporção (%) de respostas positivas na amostra para cada item ou subgrupo de alimentos ultraprocessados também foi apresentada, bem como a distribuição da amostra de acordo com cada pontuação do escore.

Para avaliar a capacidade do escore em refletir a participação dos alimentos ultraprocessados na alimentação das crianças, primeiramente utilizou-se regressão linear para avaliar a média de calorias provenientes de alimentos ultraprocessados conforme a variação na pontuação do escore, expresso na sua forma original, mas com a pontuação mais alta sendo de 10 ou mais devido ao pequeno tamanho amostral em pontuações a partir de 11, além de intervalos correspondentes a quintos (aproximados) da distribuição do escore (intervalos 0–3, 4, 5–6, 7 e ≥ 8).

A avaliação do grau de concordância entre a classificação dos participantes segundo quintos aproximados do percentual de calorias de alimentos ultraprocessados e segundo os intervalos do escore foi feita a partir do índice Pabak (*prevalence-adjusted and bias-adjusted kappa*, em português, kappa ajustado para prevalência e vieses). O índice indica concordância quase perfeita quando é superior a 0,80, substancial quando fica entre 0,61 e 0,80, moderada entre 0,41 e 0,60, razoável entre 0,21 e 0,40 e fraca quando é igual ou inferior a 0,20^18^. Para isso, foram criados quintos aproximados do percentual de energia proveniente dos alimentos ultraprocessados, com base nas proporções dos quintos aproximados do escore de consumo de alimentos ultraprocessados.

Todas as análises foram realizadas no *software* Stata 17.0 e o cálculo do índice Pabak, no *software* RStudio utilizando o pacote estatístico ˜irrCAC˜, aplicando-se pesos quadráticos por meio da função “*quadratic.weights*”. Os intervalos de confiança de 95% também foram calculados no RStudio. O método de ponderação utilizado foi escolhido com o objetivo de fornecer mais peso às categorias mais próximas.

### Aspectos Éticos

O protocolo do estudo de Coorte de Nascimentos de 2015 foi revisado e aprovado pelo comitê de ética em pesquisa da Escola Superior de Educação Física, da Universidade Federal de Pelotas (acompanhamentos de 0–4 anos: 26746414.5.0000.5313; acompanhamentos dos 6 anos: 51789921.1.0000.5317) e a assinatura do Termo de Consentimento Livre e Esclarecido (TCLE) foi obtida antes de cada entrevista.

## RESULTADOS

A amostra final do estudo foi composta por 365 crianças, sendo a maioria do sexo feminino (51,8%), de cor da pele branca (66,1%), filhas de mães com idade entre 20 e 34 anos (54,3%) e com 12 anos completos ou mais de estudo (34,5%). A Tabela 1 descreve as características sociodemográficas da amostra.

**Tabela 1:** Distribuição, segundo variáveis sociodemográficas, de crianças pertencentes à Coorte de Nascimentos de Pelotas de 2015 (n = 365a).

Características	n	%
Sexo		
Masculino	176	48,2
Feminino	189	51,8
Cor da pele		
Branca	238	66,1
Preta	63	17,5
Parda	59	16,4
Idade materna (anos)		
20–34	197	54,3
35–39	89	24,5
≥ 40	77	21,2
Escolaridade materna (anos completos de estudo)		
0–4	13	4,3
5–8	94	30,6
9–11	94	30,6
≥ 12	106	34,5
Renda familiar em reais^b^		
Q1 (mais pobres)	83	23,1
Q2	90	25,0
Q3	60	16,7
Q4	57	15,8
Q5 (mais ricos)	70	19,4

Q: quintil; m: mediana

^a^ Observacoes ignoradas: 5 (1,4%) em cor da pele, 2 (0,5%) em idade materna, 58 (15,9%) em escolaridade materna e 5 (1,4%) em renda familiar.

^b^ Renda familiar em reais: Q1 (m = 900); Q2 (m = 2.000); Q3 (m = 2.950); Q4 (m = 4.500); Q5 (10.250).

A Figura apresenta a frequência de consumo no dia anterior à entrevista de cada um dos 16 subgrupos de alimentos ultraprocessados incluídos no rastreador. Duas a cada três crianças consumiram biscoito/bolacha doce, recheada ou bolo de pacote (66%) e metade da amostra consumiu bebida achocolatada ou achocolatado em pó (adicionado ao leite) (51,8%), assim como pão de forma, de cachorro-quente ou de hambúrguer (pão de pacote) (51,5%) e margarina (49,5%). Cerca de um terço das crianças consumiu balas, pirulitos, chicletes ou gelatinas (39,5%), presunto, mortadela ou salame (37,5%), suco de caixinha ou em pó, água de coco de caixinha ou xaropes (guaraná/groselha) (37,5%), salgadinho de pacote (tipo *chips*) (34%), refrigerante (qualquer tipo) (34%), iogurte com sabor ou bebida láctea (32,3%) e chocolate, sorvete/picolé ou sobremesa industrializada (31,2%). Os demais subgrupos de alimentos foram consumidos por menos de 15% das crianças da amostra. A média do percentual de alimentos ultraprocessados consumidos foi de 49,4%.

**Figura:** Frequência (%) de consumo, no dia anterior à entrevista, de alimentos incluídos no rastreador de consumo de alimentos ultraprocessados. Coorte de Nascimentos de Pelotas de 2015. (n = 365).

A distribuição do escore de consumo de alimentos ultraprocessados (número de subgrupos consumidos no dia anterior à entrevista) está descrita na Tabela 2. O escore variou entre 0 e 10 ou mais alimentos consumidos, sendo mais frequentes os escores 4, 5 e 6 (19,7%, 16,4% e 15,9%, respectivamente). O percentual médio de participação de alimentos ultraprocessados no valor calórico total da dieta, calculado com base no R24h, foi direta e significativamente associado com o aumento do escore de consumo de alimentos ultraprocessados (Tabela 2).

**Tabela 2:** Frequência de consumo de alimentos ultraprocessados, segundo o escore, em 1 dia de recordatório alimentar aos 6 anos de idade. Coorte de Nascimentos de Pelotas de 2015. (n = 365)a.

Escore de consumo de alimentos ultraprocessados	Amostra	Ingestão energética total	Participação de alimentos ultraprocessados na dieta (% da energia total)
	n (%)	Média em Kcal	Média (IC95%)
1	11 (3,0)	1.994,3	29,9 (16,3–43,6)
2	33 (9,1)	1.967,1	41,0 (33,1–48,8)
3	31 (8,5)	2.444,6	41,5 (39,4 - 55,6)
4	72 (19,7)	2.330,4	44,5 (39,2–49,8)
5	60 (16,4)	2.338,2	49,6 (43,8–55,5)
6	58 (15,9)	2.416,9	54,9 (48,9–60,8)
7	39 (10,7)	2.443,6	50,8 (43,5–58,0)
8	29 (7,9)	2.685,6	60,4 (52,0–68,8)
9	19 (5,2)	2.941,9	57,2 (46, 9–67,6)
≥ 10	13 (3,6)	2.436,7	53,9 (41,3–66,4)^a^
1–3	75 (20,5)	2.168,5	41,1 (38,8–47,3)
4	72 (19,7)	2.330,5	44,5 (39,1–49,8)
5–6	118 (32,3)	2.376,9	52,2 (48,0–56,4)
7	39 (10,7)	2.443,6	50,8 (43,5–58,0)
≥ 8	61 (16,7)	2.712,4	58,0 (52,2–63,8)^a^

IC95%: intervalo de confianca de 95%

^a^ Subamostra de conveniencia.

^b^ Valor-p para tendencia linear < 0,001.

R2 do modelo: 0.0745

A Tabela 3 apresenta a distribuição simultânea de acordo com os quintos aproximados do escore e da participação de alimentos ultraprocessados na dieta. A partir desta distribuição da amostra, foi observada uma concordância substancial entre os intervalos do escore de consumo de alimentos ultraprocessados, obtido pelo rastreador, e os quintos aproximados da participação de alimentos ultraprocessados na dieta, calculada pelo R24h (índice Pabak de 0,65, IC95%: 0,48–0,83).

**Tabela 3:** Concordância entre a distribuição (%) de alimentos ultraprocessados em 1 dia de recordatório de 24h e quintos do escore NOVA aos 6 anos de idade. Coorte de Nascimentos de Pelotas de 2015. (n = 365).

Quintos da participação de ultraprocessados na dieta (% do total de calorias)	Quintos do escore Nova de consumo de ultraprocessados						
	1–3	4	5–6	7	≥ 8	Total	Índice PABAK^a^ (IC95%)
Q1 (< 26,6)	6	4,9	6	1,1	2,5	20,5	0,65 (0,48–0,83)
Q2 (26,7–44,1)	6	3,3	6	1,9	2,5	19,7	
Q3 (44,2–64,5)	5,8	5,8	11,8	3	6	32,4	
Q4 (64,6–74,8)	1,4	2,5	3,8	1,4	1,6	10,7	
Q5 (≥ 74,9)	1,4	3,3	4,6	3,3	4,1	16,7	
Total	20,6	19,8	32,2	10,7	16,7	100	

^a^ Índice Pabak (kappa ajustado para prevalência e viés)

## DISCUSSÃO

Este estudo apresentou um rastreador para consumo de alimentos ultraprocessados, proposto para crianças na primeira infância e avaliou a capacidade do escore, gerado a partir deste rastreador, de refletir a participação de alimentos ultraprocessados na alimentação das crianças, no contexto da Coorte de Nascimentos de 2015 da cidade de Pelotas, RS. Os resultados mostraram que o escore de consumo de alimentos ultraprocessados, obtido a partir do rastreador de consumo alimentar, apresentou uma associação direta e linear com o percentual de energia proveniente da ingestão de alimentos ultraprocessados, gerado a partir do recordatório de 24 horas. Ainda, os resultados obtidos evidenciaram uma concordância substancial entre a classificação dos participantes segundo intervalos da distribuição do escore e quintos aproximados da distribuição do percentual de alimentos ultraprocessados na alimentação.

Os resultados obtidos neste estudo vão ao encontro dos achados de estudo similar sobre uma versão do escore utilizado para adultos^19^, indicando ser possível refletir a participação de alimentos ultraprocessados na alimentação a partir dos dados obtidos pelo rastreador de consumo de ultraprocessados. Entretanto, ainda são escassos os estudos que avaliam o consumo de alimentos ultraprocessados por crianças, principalmente na primeira infância, período que compreende de zero a seis anos de idade, provavelmente pela dificuldade em medir o consumo desses alimentos, sem que sejam necessários maiores recursos financeiros e humanos para coleta de dados.

Um estudo realizado na Coorte de Nascimentos de 2015^20^, aos dois anos de idade, mostrou que cerca de 2% das crianças acompanhadas (n = 4.275) não consumiam nenhum subgrupo de alimentos ultraprocessados, habitualmente. No presente estudo, realizado com uma subamostra da mesma Coorte, todas as crianças acompanhadas consumiram ao menos um subgrupo de alimentos ultraprocessados. Outro estudo realizado na mesma Coorte avaliou o consumo de alimentos ultraprocessados aos dois e aos quatro anos de idade e mostrou que as proporções de consumo de oito dos nove subgrupos de alimentos avaliados aumentaram conforme a idade^21^. Este cenário de alto consumo é preocupante, pois, segundo recomendações dos guias alimentares para menores de dois anos^22^ e para a população brasileira^23^, os alimentos ultraprocessados não devem ser consumidos antes dos primeiros dois anos e devem ser evitados nas demais fases da vida. Nesse sentido, destaca-se a importância de novos instrumentos rápidos, curtos e fáceis de serem aplicados, que facilitem a coleta de dados alimentares e permitam que mais estudos sobre o consumo de alimentos ultraprocessados sejam realizados.

A média do percentual de alimentos ultraprocessados consumidos neste estudo foi de 49,4%. Possivelmente, o alto consumo desses alimentos pela amostra estudada não permitiu que os intervalos do escore de consumo de alimentos ultraprocessados fossem bem determinados, mostrando uma melhor concordância entre os métodos no terceiro quintil. Entretanto, apesar das porcentagens concordantes não terem sido tão altas, há concordância também nos quintos adjacentes, corroborando, portanto, a concordância substancial encontrada entre os métodos.

Este estudo possui uma limitação no que diz respeito ao rastreador de consumo de alimentos ultraprocessados. O instrumento foi construído com base nos instrumentos utilizados no Vigitel^10^ e Nutrinet Brasil^11^, ambos propostos com base nos dados da POF^13^para adultos. Apesar de ter sido baseado no rastreador Nova de consumo de alimentos ultraprocessados (NovaScreener)^7^, nosso questionário não teve a mesma metodologia, visto que não possuímos dados de inquéritos nacionais específicos para crianças na faixa etária avaliada. Outra limitação é que, apesar do tamanho de amostra suficiente para testar a concordância entre dois instrumentos^24^ (n=365), a subamostra de uma coorte de nascimentos não permitiu avaliar a capacidade do instrumento quanto a subgrupos da população, a partir de variáveis como renda familiar e escolaridade materna, por exemplo.

## CONCLUSÕES

O escore de consumo de alimentos ultraprocessados, obtido a partir de um rastreador de consumo de alimentos ultraprocessados, um instrumento prático e ágil, é capaz de refletir a participação de alimentos ultraprocessados na dieta das crianças, no que diz respeito à Coorte de Nascimentos de Pelotas de 2015. Outros estudos que avaliem este ou outros rastreadores do consumo de alimentos ultraprocessados em amostras de crianças com representatividade nacional serão importantes para fortalecer o uso dessas ferramentas na população infantil.
