# Supplementary material for: Erratum
Source: Rev Saude Publica. 2025 Jun 11;59:e5err. doi: 10.11606/s1518-8787.2025059005816err (PMC12211765; doi:10.11606/s1518-8787.2025059005816err)
Supplement: Translation

# Translation: Errata

No artigo Descrição e avaliação de um escore de consumo de alimentos ultraprocessados para crianças, com número de DOI https://doi.org/10.11606/s1518-8787.2025059005816, publicado na Revista de Saúde Pública, v. 59, e5 na página 11.

**Onde se lê:**

11. NUTRINET Brasil. Relatório do estudo Nutrinet Brasil: avaliação do impacto da alimentação na saúde dos brasileiros. São Paulo: Universidade de São Paulo; 2021 [citado 1 out. 2022]. Disponível em: http://www.nutrinetsaude.com.br/relatorio2021.

**Leia-se:**

11. Nutrinet Brasil. Nutrinet Brasil [Internet]. São Paulo: Faculdade de Saúde Pública da USP; 2025 [citado 1 out. 2022]. Disponível em: https://nutrinetbrasil.fsp.usp.br/
